# Reliability and validity of remote *Life Space Assessment* : LSA in persons with chronic stroke

**DOI:** 10.1055/s-0044-1779297

**Published:** 2024-02-07

**Authors:** Nayara Kelly de Oliveira, Laura Helen Cerqueira Gomes dos Santos, Gabriela Cristina dos Reis, Natalia Duarte Pereira

**Affiliations:** 1 Universidade Federal de São Carlos, São Carlos SP, Brazil.

**Keywords:** Stroke, Reproducibility of Results, Self-Report, Acidente Vascular Cerebral, Reprodutibilidade dos Testes, Autorrelato

## Abstract

**Background** StepWatch Activity Monitor (SAM) is used to measure the mobility of chronic hemiparetic patients and the Life Space Assessment (LSA) scale was developed to assess the displacement of hemiparetic patients in different contexts through self-reporting. Studies that apply the LSA remotely and correlate it with the number of steps measured by the SAM were not found.

**Objective** To evaluate the measurement properties of the LSA applied remotely and to evaluate the correlation between the LSA scale score and the number of steps measured by the SAM in post-stroke chronic hemiparetic patients.

**Methods** Nineteen patients participated in the study. The LSA scale was applied remotely and later, face to face. The SAM measured the steps taken by the participants over a period of three consecutive days. The correlation between the LSA and the SAM was performed using Pearson's correlation. The measurement properties calculated of remote LSA were the intraclass correlation coefficient (ICC), Cronbrach's alpha, standard error of measurement (SEM), and smallest real difference (SRD).

**Results** The reproducibility of the LSA scale between remote and face-to-face applications was considered excellent with ICC = 0.85 (IC 95% 0.62-0.94); SEM = 8.4; SRD = 23.2, and Cronbach's alpha = 0.85. The correlation between SAM and LSA was positive, considered moderate (r = 0.51) and significant (p = 0.025).

**Conclusion** The LSA is a reproducible measure for post-stroke chronic hemiparetic patients even if applied remotely and can be used as a remote measurement for mobility in a real-world environment for people with chronic hemiparesis after stroke.

## Resumo

**Antecedentes** O StepWatch Activity Monitor (SAM) é utilizado para medir a mobilidade de pacientes hemiparéticos crônicos e a escala Life Space Assessment (LSA) avalia o deslocamento de pacientes hemiparéticos em diferentes contextos por meio de autorrelato. Não foram encontrados estudos que tenham aplicado a LSA remotamente nem que a correlacionam com o número de passos mensurados pelo SAM.

**Objetivo** Avaliar as propriedades de medida da LSA aplicada remotamente e avaliar a correlação entre o escore da escala LSA e o número de passos mensurados pelo SAM em pacientes com hemiparesia crônica pós-AVC.

**Métodos** Dezenove participantes responderam a LSA remotamente e, posteriormente, presencialmente. O SAM mediu os passos dados pelos participantes durante um período de três dias consecutivos. A correlação entre a LSA e o SAM foi realizada por meio da correlação de Pearson. As propriedades de medida calculadas da LSA aplicada remotamente foram o coeficiente de correlação intraclasse (ICC), alfa de Cronbrach, erro do padrão de medida (SEM) e menor diferença real (SRD).

**Resultados** A reprodutibilidade da escala LSA entre as aplicações remotas e presenciais foi considerada excelente com ICC = 0,85 (IC 95% 0,62-0,94); SEM = 8,4; SRD = 23,2 e alfa de Cronbrach = 0,85. A correlação entre SAM e a LSA foi positiva, considerada moderada (r = 0,51) e significativa (p= 0,025).

**Conclusão** A LSA é uma medida reprodutível para pacientes hemiparéticos crônicos pós-AVC mesmo se aplicada remotamente e pode ser usada como uma medida remota de mobilidade em ambiente real para pessoas com hemiparesia crônica após AVC.

## INTRODUCTION

Stroke causes neurological sequelae, with hemiparesis being the primary and/or most common. Hemiparesis is the decrease in motor and sensory function of the lower and upper limbs on one side of the body. 1 Approximately 70% of stroke survivors regain ambulation at home, however, they remain dependent on family and community for locomotion outdoors. 2 Thus, the decrease in mobility directly impacts social participation, autonomy, and quality of life in hemiparetic patients, being that it covers aspects such as movement to change the position or location of the body, transporting objects from one place to another, walking, running or going up/down stairs and if you use different forms of transport. 3 4 5 6 Therefore, measuring the mobility of chronic hemiparetic patients after stroke in the real-world environment is an important outcome for rehabilitation, in addition to facilitating personalized management and behavioral change. 7

To measure the mobility of chronic hemiparetic patients after stroke in a real-world environment, physical activity monitors with validity and reliability are used for the continuous recording of the number of steps people with stroke, living in the community, take. 8 One of these validated monitors for hemiparetic patients is the StepWatch Activity Monitor (SAM), which is an accelerometer and microprocessor that has a wide variety of measuring capabilities, with the total step count being the most used currently. 9

Despite being widely used for research, 10 11 12 13 some barriers to the clinical use of SAM have been reported, such as cost and not providing step counts promptly. Thus, a prolonged period of use of the SAM is necessary for the data to be recorded and at least two face-to-face meetings for placement and removal of the device in the patient's lower limb. In addition, some training on the part of the evaluator for data extraction and interpretation is recommended. 14

On the other hand, the Life Space Assessment (LSA) scale was developed for the assessment and monitoring of the movement of hemiparetic patients in different contexts and environments through self-reporting. This questionnaire assesses mobility, measuring the distance covered, independence, and weekly frequency of displacement over a period of one month. 4 15 Although concurrent validity with instruments such as the Timed Up and GO (TUG), Postural Assessment Scale (PASS), and Rivermead Mobility Index have already been reported, 16 no studies have been found yet that apply to the remote LSA, nor the correlation with the number of steps measured by the SAM. The remote application of the LSA would be clinically viable in relation to the SAM since it has no costs and does not require prior training to be applied.

Thus, the objectives of this study were to evaluate the measurement properties of the LSA applied remotely and to evaluate the correlation between the LSA scale score and the number of steps measured by the SAM in post-stroke chronic hemiparetic patients. The properties calculated of remote LSA were the intraclass correlation coefficient (ICC), Cronbach's alpha, standard error of measurement (SEM), and smallest real difference (SRD).

## METHODS

### Participants

Nineteen post-stroke chronic hemiparetic individuals aged 57.9 ± 11.6 years participated in this study. who met the inclusion criteria:

- People of both genders aged 18 years or older and who received a diagnosis of chronic hemiparesis after stroke;
- Ability to independently walk at least 8 meters, 3 times a day, with or without the use of assistive devices 17 ;
- Minimum score (17 points) on the Mini-Exam questionnaire mental state (MMSE) 18 ; and
- People who did not have other previous neurological, orthopedic, and/or respiratory disorders unrelated to the stroke.

To characterize the sample, sociodemographic and physical data were collected ( Table 1 ). This study followed all the ethical recommendations established in Resolution 466/12 of the National Health Council, being approved by the Research Ethics Committee of the Federal University of São Carlos (Report 25081219.2.0000.5504).

**Table 1:** Sociodemographic data of the sample (n = 19)

		n	Average (SD)
Age		19	57.9 (11.6)
Sex	Feminine (%)	8 (42.2)	
	Masculine (%)	11 (57.8)	
MMSE		19	23.5 (3.5)
Chronicity (months)		19	72.1 (74.7)
SAM active time (min)		19	263.8 (155.6)

### Outcome measurements

#### *Life Space Assessment*

The Life Space Assessment (LSA) scale was applied to estimate the usual pattern of mobility in living spaces. The LSA score ranges from 0 to 120 points, where 0 indicates an individual restricted to the room where they sleep and 120 indicates an individual who manages to leave the city without the aid of devices or another person. The LSA was translated and adapted into Portuguese by Curcio et al. (2013), while being used in a population of elderly Brazilians and called the Brazilian version of the Life Space Assessment – Evaluation of the Space of Life. 19 Estima et al. (2015) carried out the reproducibility for the face-to-face application in hemiparetic patients, with the ICC of 0.98. 16

#### *Activity monitor*

The StepWatch TM (SAM, Modus Health, Edmons, WA, USA) is a valid and reliable tool to investigate post-stroke ambulatory activity, as it provides diverse information from the average number of steps per day (steps/day) to cadences at different intensities (steps/min) 8 . Based on the most accurate inertial sensors and widely used to count the steps of individuals with atypical, slow gait, or with prosthetic lower limbs, the SAM uses a combination of acceleration, position, and time to detect steps, in addition to being calibrated to the individual which is based on the individual's height and gait pattern.

### Procedures

After the selection and characterization of the participants, data was collected from the LSA scale; via telephone and later in person during a visit to the participant's home. After collecting the LSA data, the number of steps taken by the participants in their real-world environment was measured using the SAM accelerometer and collected. The researcher went to the residence of the selected individuals and placed the SAM on the non-paretic ankle, just above the lateral malleolus, and calibrated it for the height and walking characteristics of the participants, as recommended by the manufacturer. This placement provides more reliable step count data in adults with or without assistive devices than when placed on the hip or chest, and in addition, causes little discomfort or dislocation during usual activity. 20 Participants were instructed to use the equipment for 3 consecutive days for a minimum period of 10 hours per day. In addition, participants received instructions to write down, in an Activity Diary, all the tasks they performed during the day to verify the veracity of the data captured by the SAM. Finally, during sleep and bathing, it was recommended to remove the device, which was recorded as “non-use time” and these hours were not counted. 21

### Statistical analysis

To validate the LSA, the correlation between the total LSA score and the number of steps measured by the SAM was performed using Pearson's correlation test. The correlation was considered very low if it reached values < 0.26, low with values between 0.26 and 0.49, moderate for values between 0.50 and 0.69, high in the range of 0.70 and 0.89 or very high with values between 0.90 and -1.00. 22 To assess the reproducibility of the LSA, applied in person and remotely, the intraclass correlation coefficient (ICC) was used. ICC less than 0.4 was considered poor; satisfactory 0.4 ≤ ICC < 0.75 and excellent ICC ≥ 0.75. 23 Cronbach's alpha was calculated for each of the scores, values between 0.70 and 0.95, being considered high. The SEM was also calculated using the formula: SEM = SD√(1-ICC). From such values, the SRD was calculated (SRD = 1.96SEM√2). 24 25

## RESULTS

The participants had an average of 4.4h (± 2.6) of active time per day, which would correspond to the commuting time recorded over the period. The data that characterize the sample can be found in Table 1 .

Data from the LSA scores, applied in person and remotely, are described in Table 2 , as well as the difference between the assessments. The reproducibility of the LSA scale between remote and face-to-face applications was considered excellent with ICC = 0.85 with a 95% confidence interval of 0.62–0.94. In addition, the standard error value of the measurement was SEM = 8.4; SRD = 23.2 and Cronbach's alpha = 0.85.

**Table 2:** Summary of LSA remote and face-to-face application scores

	On-site LSA	Remote LSA	Difference from mean	Difference percentage (%)
Level 1	7.12 (1.2)	6.6 (2)	0.52	0.4
Level 2	12.5 (3.7)	12.5 (4.3)	0	0
Level 3	13.8 (8.9)	12.9 (8)	0.9	0.7
Level 4	14.2 (8.1)	11.1 (9.5)	3.1	2.6
Level 5	3.4 (4.8)	2.1 (4.5)	1.3	1
Total score	45.4 (21.7)	51.7 (18.3)	6.3	5.2

**Figure 1:**
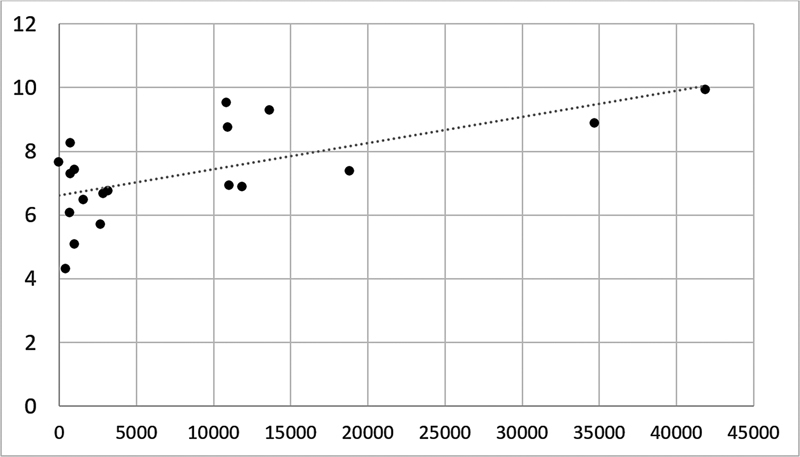
Pearson's correlation between LSA and SAM.

Pearson's correlation between the mean number of steps measured by the SAM with the total LSA score was positive, considered moderate (r = 0.51) and significant (p= 0.025). Figure 1 shows the dispersion between the SAM and LSA variables

**Figure 2:**
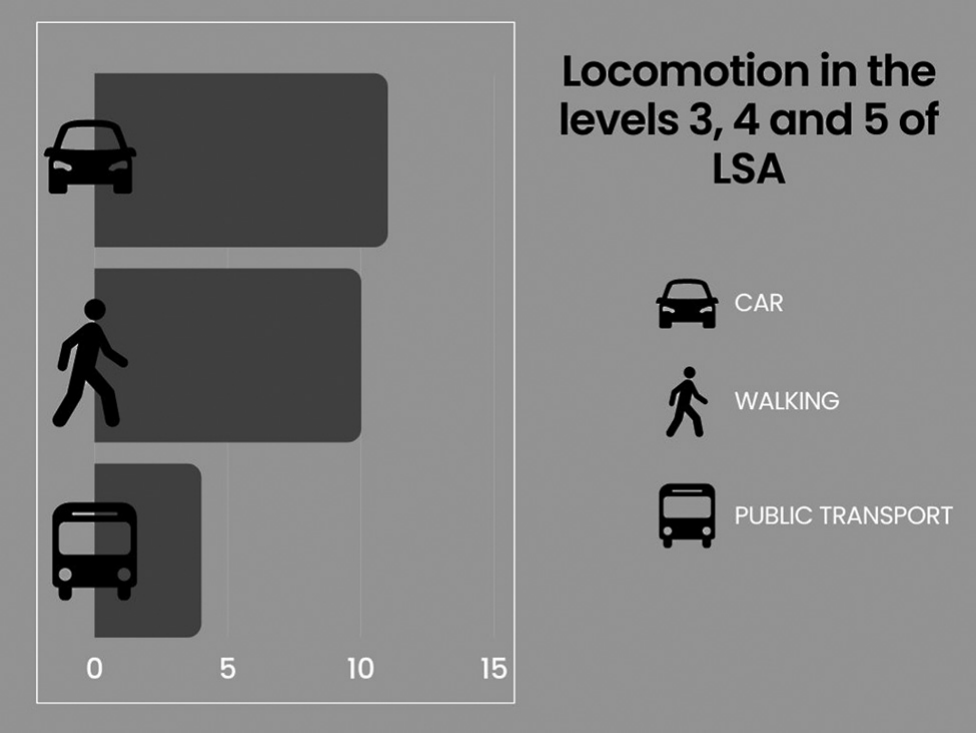
Qualitative summary of activities recorded in the SAM diary by participants, highlighting the means of transportation used.

Figure 2 shows the summary of the qualitative information collected from the activity diary. The participants completed it by inputting the activities they carried out on the three consecutive days in which they used the SAM, highlighting the places they went to and the means of transportation they used.

## DISCUSSION

This study verified the reproducibility and validity of the Life Space Assessment (LSA) scale applied remotely through the correlation of the LSA and the StepWatch activity monitor (SAM). The results show excellent reliability between the face-to-face and remote LSA measures and a positive and moderate correlation between the LSA and SAM data.

With the increased use of telehealth and the development of computational technologies, 26 27 many instruments validated only for face-to-face applications have been adapted for remote applications. Thus, there is a need to study the measurement properties of this new application format 28 and, as in the present study, other research groups have obtained, through research, the measurement properties for the remote application of assessment instruments in the stroke population. Some examples are the E-Work Life Scale (EWL) and the Fugl-Meyer, which when applied remotely remained as reliable as the LSA. 29 30 The ICC values found between the remote and face-to-face LSA were considered excellent and are close to other LSA reliability studies among face-to-face evaluators. 4 In addition, the difference in the mean total score between face-to-face and remote applications of the LSA was smaller than the SEM and SRD values. This indicates that the average variation between the face-to-face and remote LSA measurements is less than the measurement error.

The increase in recent studies to validate the application of remote instruments assists in reducing the cost of transporting people with decreased mobility for evaluation. 29 This becomes extremely relevant when the assessed construct is exactly the mobility of people with motor disabilities such as hemiparetic patients. Most instruments validated in the literature for measuring mobility in post-stroke chronic hemiparetic patients are activity monitors and face-to-face functional tests; among these, the most used are SAM and TUG, respectively. 14 31 The correlation of the LSA with the SAM then presents an alternative for the clinical evaluation of aspects of mobility, minimizing the need for face-to-face tests.

Despite the previously mentioned benefits regarding the remote application of the LSA, there are some barriers related to the hemiparetic population, since one of the possible sequelae that the stroke can cause is aphasia, which in some cases makes it difficult to communicate exclusively verbally, such as in a telephone call. Limitations of this study included a relatively small sample size which may limit result generalization. However, the reliability was considered excellent, further studies are necessary, with a larger number of participants. Furthermore, the present study did not make any adaptations for application via video conference, which could enable people with expression aphasia to respond using non-verbal communication.

In conclusion, the LSA is a reproducible measure for post-stroke chronic hemiparetic even if applied remotely. In addition, there is a positive and moderate correlation between the LSA score and the number of steps measured by the SAM. However, the reproducibility of this study still requires further research before LSA can be used as a remote measure of mobility in a real-world setting for people with chronic post-stroke hemiparesis.
